# Post-infectious bronchiolitis obliterans in children: a review of 42 cases

**DOI:** 10.1186/1471-2431-14-238

**Published:** 2014-09-25

**Authors:** Ya-Nan Li, Li Liu, Hong-Mei Qiao, Hang Cheng, Huan-Ji Cheng

**Affiliations:** Department of Pediatrics, The First Hospital of Jilin University, Changchun, 130021 PR China; Institute of Pediatrics, The First Hospital of Jilin University, Changchun, 130021 PR China

**Keywords:** Children, HRCT, Post-infectious bronchiolitis obliterans (PIBO), Pulmonary function testing

## Abstract

**Background:**

This study aimed to describe the clinical characteristics, radiological features and outcomes of 42 children with post-infectious bronchiolitis obliterans (PIBO).

**Methods:**

Forty-two children diagnosed with PIBO were prospectively studied at the First Hospital of Jilin University in northern China between January, 2008 and January, 2013. Their clinical characteristics, lung high resolution computed tomography (HRCT) findings and pulmonary function tests were reported.

**Results:**

In children with PIBO, adenovirus was the most common etiologic agent (21/42), followed by *Mycoplasma pneumoniae (M. pneumoniae)*. All of the patients presented with repeated wheezing and tachypnea. In addition, 22 patients required intensive management, while six patients required home oxygen therapy. HRCT findings were consistent with the PIBO diagnosis in all of the patients. Pulmonary function testing was useful in evaluating therapeutic responses. Systemic steroids combined with azithromycin were effective for PIBO treatment.

**Conclusions:**

Severe adenovirus bronchiolitis and *M. pneumoniae* infections have a higher risk of development for PIBO. HRCT and pulmonary function testing are useful in the diagnosis of PIBO. The degree of airway obstruction did not differ significantly between adenovirus and *M. pneumoniae*. A combination of steroids and azithromycin offers some benefit in treating these patients.

## Background

Bronchiolitis obliterans (BO) was first reported and named in 1901 as small airway injury-related chronic inflammation airflow obstruction syndrome [1]. BO can be caused by various etiological factors, including infection, organ transplantation and exposure to toxic fumes. BO results in bronchiolar epithelial cell and subepithelial structural damage and inflammation [2]. Furthermore, improper small airway repair with intraluminal scarring can aggravate inflammation [3]. Post-infectious bronchiolitis obliterans (PIBO) is especially common in children [2, 4]. The incidence of PIBO in severe adenovirus pneumonia is high. One study found that 47.4% of children hospitalized with adenovirus pneumonia developed BO during 5 years of follow-up [5]. However, the pathogenesis of PIBO is still not completely understood. In addition, there is no effective treatment and it has a poor prognosis [6, 7]. PIBO has a huge psychological and economic burden on the families of affected children, as well as the community. The clinical manifestations, including repeated coughing, wheezing and shortness of breath following activities, are nonspecific. In addition, PIBO progression varies, chest x-ray and laboratory tests are not sufficient to diagnose the disease, and PIBO is often misdiagnosed as asthma, pneumonia and other diseases. Despite the great impact of PIBO on children, clinical data is scarce and most clinicians have limited awareness of the disease. Early diagnosis of PIBO is difficult, and the effect of therapy is very poor. Therefore, we prospectively analyzed the clinical characteristics, radiological features and outcome of 42 children with PIBO in order to describe key aspects of the diagnosis and treatment of PIBO.

## Methods

This prospective study included 42 PIBO cases from January, 2008 to January, 2013 at the First Hospital of Jilin University. All of the children included in the study were less than 14 years of age, were diagnosed with PIBO in our hospital, had an acute lower respiratory infection (ALRI) with an infective pathogen identified, and had more than 6-months of follow-up. The diagnosis of PIBO was based on previously published criteria [8–10] and met all of the following criteria: 1) Documented ALRI in otherwise healthy children with exercise intolerance, and repeated or continuous wheezing, coughing and tachypnea; 2) Mosaic ground-glass patterns, bronchiectasia or pulmonary atelectasis on high resolution computed tomography (HRCT); 3) Persistent obstructive pattern on pulmonary function tests after the acute event in older children; and 4) Exclusion of other diseases, such as asthma, primary ciliary dyskinesia, cystic fibrosis, foreign body aspiration, tuberculosis, AIDS and other immune function defects. All of the patients had follow-up visits at 1, 3 and 6 months after being diagnosed with PIBO and underwent pulmonary function tests. In addition, we recorded the clinical symptoms for all of the patients. The Ethics Committee of the First Hospital of Jilin University approved the study protocol. The parents or legal guardians of the participants gave written informed consent.

All patients underwent HRCT at the time of diagnosis and underwent pulmonary function testing using Master Screen Paed (Jaeger Company, Wurzburg, Germany) at the time of diagnosis and during follow-up at 1, 3 and 6 months. Airway obstruction was defined using tidal breathing analysis (in children aged <3 years) indicating an t_PTEF_% t_E_<40% and V_PEF_%V_E_<40% or impulse oscillometry (in children aged 3–6 years) showing Z5 (magnitude of respiratory system impedance at 5 Hz), R5 (total respiratory resistance at 5 Hz) and X5(distal capacitive reactance at 5 Hz) >120% of the predicted value. HRCT scans were obtained using a Somaton Sensation Cardiac 64 (Siemens AG, Forchheim, Germany) CT scanner. The scans covered an area from the upper margin of the shoulder to the lower end of the liver. The specifications of the scans included a 0.33 s rotation time, 1.2 spiral distance, 0.6 mm collimation, 0.6 mm slice thickness, 120 kV voltage, and 120 mA tube-current. After the scan, images were obtained using a 1 mm reconstruction increment and a 0.6 mm reconstruction distance. Each HRCT examination consisted of five images, which were obtained at the levels of the aortic arch, midway between the aortic arch and tracheal carina, tracheal carina, midway between the tracheal carina and the right hemidiaphragm, and 1 cm above the right hemidiaphragm.

All of the reconstructed images were loaded into the HRCT work station. The radiological findings were interpreted by three senior radiologists. A consensus through discussion was obtained whenever there was a discrepancy. Mosaic pattern, bronchiectasis and peribronchial thickening are considered as the most common CT signs in PIBO, and the latter two features are used as diagnostic criteria for PIBO [11].

Age at the initial acute injury, days of initial hospitalization, oxygen requirements and pulmonary function tests were evaluated. If a patient was admitted to the hospital in an acute state, the etiological agents were investigated by serology. All of the patients had documented results of the etiologic agent causing the original lower respiratory tract infection. Indirect immunofluorescence testing was used to diagnose infection with *Influenza A virus*-IgM, *Influenza B virus*-IgM, *M. pneumoniae*-IgM, *Chlamydia pneumoniae* (*C. pneumoniae*)-IgM, *Legionella pneumophila*-IgM, Q fever rickettsia-IgM, adenovirus (ADV)-IgM, respiratory syncytial virus (RSV)-IgM, and parainfluenza virus-IgM. Based on serological test results, we classified the 42 patients into three groups: 1. Adeno-associated BO (G1); 2. *M. pneumoniae* -associated BO (G2); and 3. Other agent BO (G3). A flexible fiberoptic bronchoscope (FOB; Olympus LF-2) was used in 20 patients to view their airways and to check for any abnormalities before diagnosis.

The treatment protocol for PIBO included oral prednisone and azithromycin. The dose of prednisone was initially 1.5 mg/kg/day, which was given in divided doses. Four weeks later the dose was gradually decreased to a single early morning dose of 0.5 mg/kg/day. The total course of steroids was 6 to 9 months. Azithromycin was given orally at 5 mg/kg/day, once daily for 3 consecutive days per week for 6 months. All of the patients were evaluated 6 months following the initiation of treatment. The effect of treatment was categorized as follows: 1) Effective, a reduction in wheezing by more than 50%, increased activity (tolerated daily physical activity such as climbing stairs, walking, etc.), and lung function remained stable (the rate of decline of pulmonary function was less than 10% when compared with the initial test); and 2) Ineffective, defined as no improvements in wheezing, decreased activity and worsened lung function (the rate of decline of pulmonary function was more than 10% when compared with the initial test).

### Statistics

Results including age at injury, age at diagnosis and hospitalization were expressed as means and ranges, pulmonary function test results were expressed as mean ± SD. Independent sample t-tests were used to compare continuous variables and Fishers exact tests were used to compare categorical variables. SPSS 17.0 was used for all statistical analyses. The level of statistical significance was set at p <0.05.

## Results

**Figure 1:**
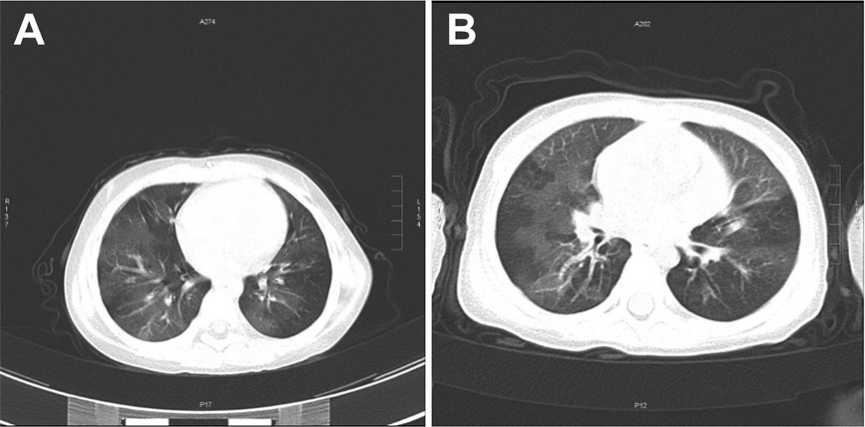
**HRCT scan shows a mosaic pattern and bronchiectasis in a 2-year-old boy (A) and 3- year-old girl (B) with PIBO, respectively.**

A total of 42 children diagnosed with PIBO based on HRCT and clinical features were identified. Table 1 shows the distribution of HRCT findings, and Figure 1 shows HRCT findings of mosaic ground-glass patterns and bronchiectasis in two representative cases. The clinical characteristics of all of the patients with PIBO are presented in Table 2. Among the 42 patients, 29 were boys and 13 were girls (male/female ratio was 2.2:1). The mean age at initial pulmonary injury was 1.46 (range 0.6–3.8) years, and the median age at diagnosis of PIBO was 2.32 (range 0.8–5.7) years. The mean hospital stay was 27.8 (range, 14–73) days. There were no statistical differences with respect to age, gender and hospital stay between the groups. In addition, the long-term home oxygenation therapy (LTOT) rate was not significantly different between G1 and G2, G1 and G3, and G2 and G3.

**Table 1:** **Chest HRCT findings in 42 children**

HRCT findings	n	%
Mosaic pattern	41	97.6
Bronchiectasis	35	83.3
Peribronchial thickening	28	66.7

**Table 2:** **Clinical characteristics of 42 cases and the three subgroups: adeno-associated BO group,** ***M. pneumoniae*** **-associated BO group and other agent BO group**

	Total cases, n = 42	G1, n = 21	G2, n = 10	G3, n = 11	***P*** ^1^	***P*** ^2^	***P*** ^3^
Age at injury, years, (mean and ranges)	1.38 (0.6–3.8)	1.30 (0.6–3.4)	1.51 (0.7–3.8)	1.43 (0.7–3.7)	0.13	0.09	0.16
Gender (%males)	69.0	66.7	60.0	81.8	1.00	0.44	0.36
Age (years) at diagnosis (mean and range)	2.18 (0.8–5.7)	1.87 (0.8–5.4)	2.60 (1.0–5.7)	2.42 (1.1–5.1)	0.27	0.34	0.12
Hospitalization (days) (mean and range)	30.3 (14–73)	35.2 (16–73)	28.5 (18–64)	22.4 (14–47)	0.11	0.17	0.29
LTOT, n (% of cases)	6(14.3)	4 (19.0)	1 (10.0)	1(9.1)	1.00	0.63	1.00
Effective, n (% of cases)	36(85.7)	19 (90.5)	9 (90.0)	8 (72.7)	1.00	0.31	0.58

Note: *P* ^1^ = comparing G1 with G2; *P* ^2^ = comparing G1 with G3; *P* ^3^ = comparing G2 with G3. LTOT, long term home oxygen therapy. Independent sample t-tests were used to compare continuous variables, including age at injury, age at diagnosis and hospitalization. Fishers’s exact tests were used to compare categorical variables, including gender, LTOT and effectiveness.

**Figure 2:**
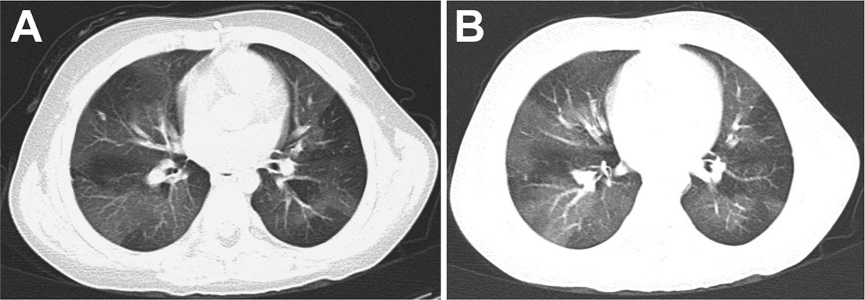
**Pre-treatment (A) and post-treatment (B) HRCT scans of the lung in a 5-year-old boy.** There was no significant improvement following treatment.

Initial presentations included fever in 29 (69.0%) patients, cough in 28(84.8%) patients, and tachypnea in 24 (57.1%) patients. Physical examination revealed crackles in 28 (66.7%) patients, wheezing in 37 (88.1%) patients, and chest retractions in 18 (42.9%) patients. All of the patients had persistent dyspneic respirations and wheezing from the time of the initial lung infection. During follow-up, three children (7.1%) required conventional mechanical ventilation. The initial pulmonary function tests revealed increased airway resistance in all of the patients. Fiberoptic bronchoscopy was performed in 20 patients. Six patients had normal findings, two patients had laryngomalacia, and 12 patients had bronchial mucosal inflammation. The tidal breathing analysis revealed a decreased t_PTEF_% t_E_ (18.2 ± 0.26%, normal: more than 40%) or V_PEF_%V_E_ (21.7 ± 0.32%, normal: more than 40%), suggesting obstructive airway dysfunction. The impulse oscillometry results showed abnormally increased values for Z5 (147.5 ± 19.3% of the predicted value, normal: less than 120% of the predicted value), R5 (140.4 ± 12.8% of the predicted value, normal: less than 120% of the predicted value) and X5 (226.5 ± 13.4% of the predicted value, normal: less than 120% of the predicted value), suggesting increased peripheral airway resistance. Adenovirus was detected in 21 patients, including one patient who had simultaneous *Influenza A virus*-IgM positivity. *M. pneumoniae* specific IgM was detected in 10 patients, RSV specific IgM was positive in 6 patients, *C. pneumoniae*-IgM was positive in 2 patients, and *Influenza B virus*-IgM, *Influenza A virus*-IgM and *Legionella pneumophila*-IgM was positive in one patient respectively. The degree of airway obstruction did not differ significantly between etiologic agents (Table 3). All of the patients received oral corticosteroids. Twenty-six patients (61.9%) required intensive care management and 17 (40.5%) patients required mechanical ventilation due to progressive dyspnea and respiratory failure in the acute phase. Intravenous corticosteroid therapy was given to 28 (66.7%) patients during the acute stage, while six (14.3%) patients required LTOT. In addition, 31 (73.8%) patients required frequent re-admissions due to acute exacerbations of respiratory symptoms. Furthermore, at least six months of supportive treatment was needed for clinical improvement in 36 cases (effective treatment). Treatment was considered effective in 36 (85.7%) patients and ineffective in six (14.3%) cases at the end of six months of therapy. There was no significant improvement on the CT scan images post treatment when compared with pretreatment images (Figure 2).

**Table 3:** **Pulmonary function test results of children with PIBO at the time of diagnosis (mean ± SD)**

Pulmonary function tests	Test index	Estimated value (% Predicted)						
		Total cases, n = 42	G1, n = 21	G2, n = 10	G3, n = 11	***P*** ^1^	***P*** ^2^	***P*** ^3^
Tidal breathing analysis	t_PTEF_%t_E_	(23.9 ± 4.6)%	(22.9 ± 4.8)%	(25.4 ± 6.2)%	(24.6 ± 6.5)%	0.23	0.41	0.78
	V_PEF_%V_E_	(20.5 ± 3.9)%	(19.4 ± 4.1)%	(20.8 ± 4.7)%	(22.4 ± 5.1)%	0.40	0.08	0.47
Impulse oscillometry		Total cases, n = 42	G1, n = 8	G2, n = 4	G3, n = 4	*P* ^1^	*P* ^2^	*P* ^3^
	Z5	(147.5 ± 39.3)%	(153.2 ± 44.2)%	(134.9 ± 51.7)%	(148.7 ± 36.0)%	0.54	0.86	0.68
	R5	(140.4 ± 32.9)%	(137.2 ± 40.1)%	(129.5 ± 43.4)%	(157.7 ± 32.9)%	0.77	0.40	0.34
	X5	(226.5 ± 63.6)%	(189.2 ± 40.4)%	(247.7 ± 59.3)%	(279.9 ± 63.6)%	0.07	0.01	0.49

Note: tPTEF%tE: ratio of time to reach peak tidal expiratory flow to total expiratory time; VPEF%VE: ratio of volume to reach peak expiratory flow to total expiratory volume; Z5: magnitude of respiratory impedance; R5: total respiratory resistance; X5: distal capacitive reactance; FEV1: forced expiratory volume in 1 sec; PEF: peak expiratory flow. Independent sample t-tests were used to compare continuous variables, including tPTEF%tE, VPEF%VE, Z5, R5 and X5.

*P* ^1^ = comparing G1 with G2; *P* ^2^ = comparing G1 with G3; *P* ^3^ = comparing G2 with G3.

## Discussion

PIBO has been traditionally considered as a rare condition. In this study, we described 42 children with PIBO, and the general characteristics were similar to previous studies [4, 8, 9]. In our study, most of the patients suffered from lower respiratory infections during the first 2 years of life, were hospitalized, and required oxygen and other supportive care at discharge. In previous studies, the disease prevalence was observed to be higher in boys than girls [10, 12]. Our results were consistent with these studies, with a male to female ratio of 2.2:1. The pulmonary function tests suggested increased small airway resistance in all of the PIBO patients.

PIBO is most commonly associated with adenovirus infection, but other pathogens such as *M.pneumoniae* may also be associated with this condition [2, 13–15]. This was consistent with the findings in our study, indicating the need to strengthen the prevention of *M. pneumoniae* infection.

The definitive and effective treatment for PIBO is unknown. Current treatment methods include oral azithromycin and glucocorticoids, supportive care, and mechanical ventilation when there are severe breathing difficulties [12, 16]. Some benefits of long term azithromycin therapy has been found in patients with post-lung-transplant bronchiolitis obliterans, including improved forced expiratory volume in one second (FEV1 ) values and decreased airway neutrophilia and IL-8 levels [17, 18]. Mechanical ventilation is indispensable for the support of critically ill patients with respiratory insufficiency. Our results indicated that mechanical ventilation was needed in 40.5% of children in the acute phase and 7.1% of children during follow-up.

Few studies have shown definitive effects of systemic corticosteroids and oral azithromycin in the treatment of PIBO [4, 19]. Beneficial effects of azithromycin in BO have been primarily reported in adult patients who underwent lung transplants. This study showed that treatment with corticosteroids and azithromycin was effective in 85.7% of cases and ineffective in 14.3% of cases. However, we did not have a control group, and thus our study is limited. In a previous prospective study of 31 patients with 3.5 years of follow-up, the outcome of the patients included clinical remission (22.6%), persistence of respiratory symptoms and signs (67.7%), and death (9.7%) [20].

The clinical history and HRCT images were essential for the diagnosis of PIBO in this study, as well as for excluding other diseases. Open pulmonary biopsies, although considered the gold standard, is generally not required for the diagnosis of PIBO. This invasive investigation may be considered in patients who show progressive deterioration even after treatment. Pulmonary function tests play an important role in the diagnosis and therapeutic evaluation of PIBO. Our study showed that airway obstruction was present in all of the patients diagnosed with PIBO, and the degree of obstruction was not different among the three groups.

This study had some limitations. First, open lung biopsy was not obtained. Second, immunofluorescence IgM testing was used to determine the etiological agents. This method is accurate, quick and simple; however, the sensitivity and specificity is lower than that of polymerase chain reaction (PCR). Third, to achieve power of 80% and an alpha error of 0.05, the sample size of each group should be 36. However, in our study, the sample size of the three groups was 21, 11, and 10, respectively, which reduced the power of the study. Fourth, the treatments were administered at different stages of the disease. Lastly, the follow-up time limited the assessment of long term prognosis and did not exclude the confounding factor of spontaneous improvement as the child ages.

## Conclusions

In conclusion, severe adenovirus bronchiolitis and *M. pneumoniae* pneumonia appeared to have a higher risk of development of PIBO in Chinese children. Lung HRCT and pulmonary function testing contribute to the diagnosis of PIBO. Systemic steroids in combination with azithromycin may offer some benefit for PIBO patients.

## Abbreviations

PIBO: Post-infectious bronchiolitis obliterans
HRCT: High resolution computed tomography
BO: Bronchiolitis obliterans
ALRI: Acute lower respiratory infection
LTOT: Long-term home oxygenation therapy.
